# The Etiology of Leprosy

**Published:** 1881-02

**Authors:** 


The Etiology of Leprosy.—In an article by Jonathan Hutchison, Esq., F.R.S., on the etiology of true leprosy, recently published in the *Medical Press and Circular*, he says: "We find that nearly everywhere the disease is most common on the sea shore, and that when it spreads inland it generally occurs on the shores of lakes, or along the course of large rivers; we find also that fish is largely eaten where leprosy prevails." He continues: "I don't believe that any other article of diet has any influence whatever on the causation of the disease; this is so special in its character that it must have one special cause."

In confirmation of this opinion, I will state my experience, acquired while on a visit three years ago to the north shore of New Brunswick, Dominion of Canada. Here leprosy has prevailed more or less since its early settlement by the French, having been brought over with them.

At the village of Tracadie a lazaretto has been established for several years, and is under the charge of the Sisters of Charity of Montreal, and at the time of my visit contained twenty-nine inmates in various stages of the disease.

Upon inquiry I learned that there was no regular medical attendant connected with the institution, but that the nuns prescribed and dispensed the medicines themselves under some general instruction. In conversation with one of the trustees, a very intelligent gentleman, I learned that the inhabitants were very poor and that the disease was confined to the descendants of the French, living almost exclusively on fish and potatoes, prefering the former in a state af decomposition, or, as he expressed it, putrid, having acquired a taste for it similar to that of the English for high pheasant. I also learned that cancer is a very common disease among these poor people.—*Medical Record.*

